# Comparison of clinical results between trans-PRK and femtosecond LASIK for correction of high myopia

**DOI:** 10.1186/s12886-020-01515-9

**Published:** 2020-06-19

**Authors:** Jiafan Zhang, Qingqing Feng, Wenzhi Ding, Yusu Peng, Keli Long

**Affiliations:** 1 Qingdao Eye Hospital, State Key Laboratory Cultivation Base, Shandong Provincial Key Laboratory of Ophthalmology, Shandong Eye Institute, Shandong First Medical University & Shandong Academy of Medical Sciences, Qingdao, 266071 Shandong Province China; 2 Qingdao Center Hospital, the Second Affiliated Hospital of Qingdao University, Qingdao, Shandong Province China

**Keywords:** TPRK, FS-LASIK, Myopia, Visual acuity, Refraction

## Abstract

**Background:**

To compare the clinical outcomes of transepithelial photorefractive keratectomy (TPRK) with femtosecond laser-assisted in situ keratomileusis (FS-LASIK) for correction of high myopia.

**Methods:**

In this prospective, non-randomised, cohort study, 85 eyes of 46 patients treated with TPRK and 80 eyes of 42 patients treated with FS-LASIK were included. All eyes were highly myopic (spherical equivalent refraction <− 6.00 diopters). Both TPRK and FS-LASIK were performed by Schwind Amaris 750S excimer laser. Visual acuity, refraction, corneal high order aberration (HOA) and other variables were analyzed before and at 1, 3, 6, 12 months after surgery.

**Results:**

At 12 months after surgery, uncorrected logMAR distance visual acuity (UDVA) in the TPRK and FS-LASIK groups was − 0.04 ± 0.04 and − 0.01 ± 0.08, respectively (*P* = 0.039). Corrected logMAR distance visual acuity (CDVA) was − 0.06 ± 0.05 and − 0.04 ± 0.05 in both groups (*P* = 0.621). For UDVA, 86% of eyes in the TPRK group and 80% in the FS-LASIK group remained unchanged or improved one or more logMAR lines (*P* = 0.314), compared to preoperative CDVA. For CDVA, 97% of eyes in the TPRK group and 90% in the FS-LASIK group remained unchanged or improved one or more lines (*P* = 0.096), compared to preoperative CDVA. Spherical equivalent refraction was − 0.05 ± 0.39 and − 0.26 ± 0.47 in both groups (*P* = 0.030). 87% of eyes in the TPRK group and 73% in the FS-LASIK group achieved ±0.50 D target refraction (*P* = 0.019). All 85 eyes (100%) in the TPRK group and 75 eyes (92%) in the FS-LASIK group were within ±1.00 D of target (*P* = 0.003). Root mean square (RMS) of corneal total HOA and vertical coma in the TPRK group were lower compared with the FS-LASIK group (*P* < 0.001 for both variables).

**Conclusions:**

TPRK and FS-LASIK showed good safety, efficacy and predictability for correction of high myopia. Clinical outcomes of TPRK were slightly better than FS-LASIK.

## Background

The efficacy and safety of corneal refractive surgery has improved since excimer laser photorefractive keratectomy was introduced to treat refractive error in the human eye. New surgical techniques such as corneal lamellar surgery, femtosecond assisted laser in situ keratomileusis (FS-LASIK) and small incision lenticule extraction (SMILE) have emerged as the preferred procedures because of rapid visual recovery, less pain, and less corneal haze [1]. However, LASIK had some adverse outcomes, including intraoperative and late flap-related complications, corneal biomechanical instability, and iatrogenic keratectasia that were more likely to occur in high myopia [2–4]. Corneal surface laser ablation had advantages of not needing to make a flap and greater stability of postoperative corneal biomechanics than LASIK [5]. Compared with LASIK for correcting high myopia, surface ablation retained more corneal stromal tissue, thus avoiding the potential risk of keratectasia [6].

In recent years, surface ablation techniques have improved. A new procedure, transepithelial photorefractive keratectomy (TPRK), was introduced as an alternative to conventional PRK. This avoided the need for alcohol epithelial debridement or mechanical removal of the epithelium during PRK [7, 8]. TPRK requires only one-step removal of the epithelium and stroma, has no instrument contact with the cornea, takes less surgical time, less postoperative pain, faster wound healing and faster visual recovery than conventional PRK [9].

Studies have shown that TPRK, PRK or LASEK are efficient and safe methods to correct low and moderate myopia [10, 11]. However, for high myopia, the stability and predictability of correction may be reduced. Larger stromal ablation is required causing more extensive wound healing [12, 13]. Correction of high myopia remains a difficult challenge for both corneal lamellar and surface ablation, Furthermore, whether lamellar surgery or surface ablation provides the better outcome remains inconclusive.

The aim of this prospective, non-randomised, cohort study is to compare 12-months clinical outcomes of predictability, safety, efficacy and corneal high order aberration, using TPRK with FS-LASIK for high myopia.

## Methods

### Patients and study design

This prospective, non-randomised, cohort study recruited patients with high myopia (−6D or more) with or without astigmatism who attended in Qingdao Eye Hospital, Qingdao, China consecutively from January 2018 to June 2018. Patients were divided into two groups: the case group for whom TPRK was performed and the control group who received FS-LASIK. The choice of surgical procedure mainly depended on the patient’s preference (after detailed description of the procedures). To compare postoperative changes with minimal bias, both groups of patients were matched based on preoperative indices. All patients were adequately informed about the study as well as the risks and benefits of the surgery and provided signed informed consent to participate. The study protocol followed the tenets of the Declaration of Helsinki and was approved by the Ethics Committees of Institutional Review Boards.

Inclusion criteria were as follows: age over 18 years with stable refraction for at least 12 months, discontinuance of soft contact lens wear for a minimum of 1 week and rigid CL wear for at least 1 month prior to preoperative examination. Exclusion criteria were abnormal or keratoconic topography, previous ocular surgery, concurrent ocular diseases and systemic diseases that could affect corneal wound healing.

### Ocular examination

All patients received a complete eye examination including uncorrected (UDVA) and corrected (CDVA) distance visual acuity, manifest and cycloplegic refraction, slit-lamp evaluation of the anterior and posterior segment, intraocular pressure (IOP), axial length, keratometry and corneal topography (Pentacam; Oculus, Wetzlar, Germany), corneal epithelial thickness measured with anterior segment optical coherence tomography (RTVue OCT, Optovue, America), central corneal thickness (CCT) measured with an ultrasonic pachymeter (US-500, NIDEK, Japan) and ocular fundus examination. Corneal aberration was measured by the Pentacam in a dark room. High order aberration (HOA) included trefoil, coma, spherical aberration, etc. HOA were calculated with a 6.0-mm diameter of the pupil.

### Surgical procedures

In the TPRK group, all surgeries were performed with the Amaris 750S excimer laser (Schwind eye-tech-solutions, Germany). Prior to laser ablation, a wet sponge application was used to wipe corneal surface evenly, prevent uneven wetting and thus uneven ablation. The ablation zone was set to 5.9–6.3 mm and a blend zone of 1.5–2.0 mm. After laser ablation, the cornea was cooled with 10 ml chilled balanced salt solution. A soft bandage contact lens (Pure Vision, Bausch & Lomb) was applied and one drop of 0.3% tobramycin dexamethasone was instilled. All patients were instructed to use topical instillation of 0.3% tobramycin dexamethasone and 0.3% gatifloxacin qid until removal of the contact lens. Following healing of the corneal epithelium, we prescribed 0.1% fluoromethane drops qid for the first month (then reducing once a month), and 0.3% sodium hyaluronate drops qid for 4 months.

In the FS-LASIK group, the cornea flap was made with WaveLight FS200 femtosecond laser. The laser platforms were programmed to create a flap with a thickness of 110 mm and a diameter from 8.1 to 8.5 mm. After lifting the flap, ablation was performed with Amaris 750S excimer laser for a 6.0–6.3 mm optical zone and a blend zone of 1.5–2.0 mm. After surgery, all patients were prescribed 0.3% gatifloxacin drops qid for 1 week and 0.1% fluoromethane drops qid for 3 weeks (reducing once a week) and 0.3% sodium hyaluronate drops qid for 3 months. A new optional software feature named smart pulse technology (SPT) that ameliorates the stromal bed contour was introduced at Amaris 750S laser platform. SPT was used not only in TPRK group but also in FS-LASIK group.

All patients were followed up at 1 day, 3 days, 1 week, 1 month, 3 months, 6 months and 12 months postoperatively. These assessments included visual acuity (UDVA and CDVA), subjective refraction, IOP, topography, and assessment of high order aberration (HOA).

### Statistical analysis

Data were analyzed using IBM SPSS version 22.0 (IBM Inc., New York, USA). The normality of the data was verified with Kolmogorov-Smirnov test. Comparisons for the preoperative data between both groups were performed using the Mann-Whitney test for non-normally distributed data and the unpaired t test for normally distributed data. Repeated measures analysis of variance was used to evaluate whether variables were influenced by time in each group. The independent sample t-test was used to compare variables between both groups at different time points. Chi-square or Fischer-exact test was used to compare categorical variables. A *P* value of less than 0.05 was considered statistically significant.

## Results

We commenced with 48 patients with high myopia in the TPRK group and 45 patients in FS-LASIK group. Two patients in the TPRK group and three patients in the FS-LASIK group were excluded after loss to the follow-up at 12 months. Finally, 85 eyes in 46 patients receiving TPRK and 80 eyes in 42 patients receiving FS-LASIK were included in the analysis (Table 1). There were no significant differences between the two groups in terms of preoperative variables, including age, gender percentage, UDVA, CDVA, sphere, spherical equivalent refraction, IOP, CCT, flat meridian curvature, steep meridian curvature, optical zone (*P* > 0.05, except for CCT *P* = 0.05).

**Table 1:** Demographics and preoperative variables of the study groups (mean ± SD)

Parameter	TPRK	FS-LASIK	*P*
No. of eyes	85	80	
No. of patients	46	42	
Male (%)	20(43.5%)	17(40.5%)	0.776
Age (years)	25.6 ± 6.1	23.9 ± 5.5	0.161
UDVA (logMAR)	1.22 ± 0.20	1.25 ± 0.21	0.406
CDVA (logMAR)	−0.02 ± 0.04	− 0.01 ± 0.03	0.135
Sphere (D)	−7.04 ± 0.85	−7.09 ± 1.23	0.791
Range (D)	−5.50 to −9.75	− 5.00 to − 9.75	
Cylinder (D)	−1.11 ± 0.52	− 1.03 ± 0.74	0.419
Range (D)	−0.50 to −3.00	− 0.25 to − 3.00	
SER (D)	−7.59 ± 0.84	− 7.60 ± 1.21	0.947
IOP (mmHg)	15.1 ± 2.5	15.4 ± 2.5	0.448
CCT (μm)	533.0 ± 23.0	540.0 ± 22.8	0.050
K1	42.6 ± 1.6	42.6 ± 1.2	0.710
K2	43.8 ± 1.7	44.1 ± 1.5	0.255
Ablation depth (μm)	103.5 ± 7.0	106.4 ± 13.2	0.085
Optical zone (mm)	6.2 ± 0.2	6.3 ± 0.2	0.085
Corneal total HOA (μm)	0.40 ± 0.09	0.42 ± 0.09	0.276
Spherical aberration (μm)	0.19 ± 0.08	0.20 ± 0.11	0.434
Vertical coma (μm)	−0.06 ± 0.17	−0.04 ± 0.20	0.718
Horizontal coma (μm)	0.01 ± 0.13	0.02 ± 0.12	0.499

*UDVA* uncorrected distance visual acuity, *CDVA* corrected distance visual acuity, *SER* spherical equivalent refraction, *IOP* intraocular pressure, *CCT* central cornea thickness, *K1* cornea flat meridian curvature, *K2* cornea steep meridian curvature, *SD* standard deviation

### Visual acuity, efficacy and safety

**Fig. 1:**
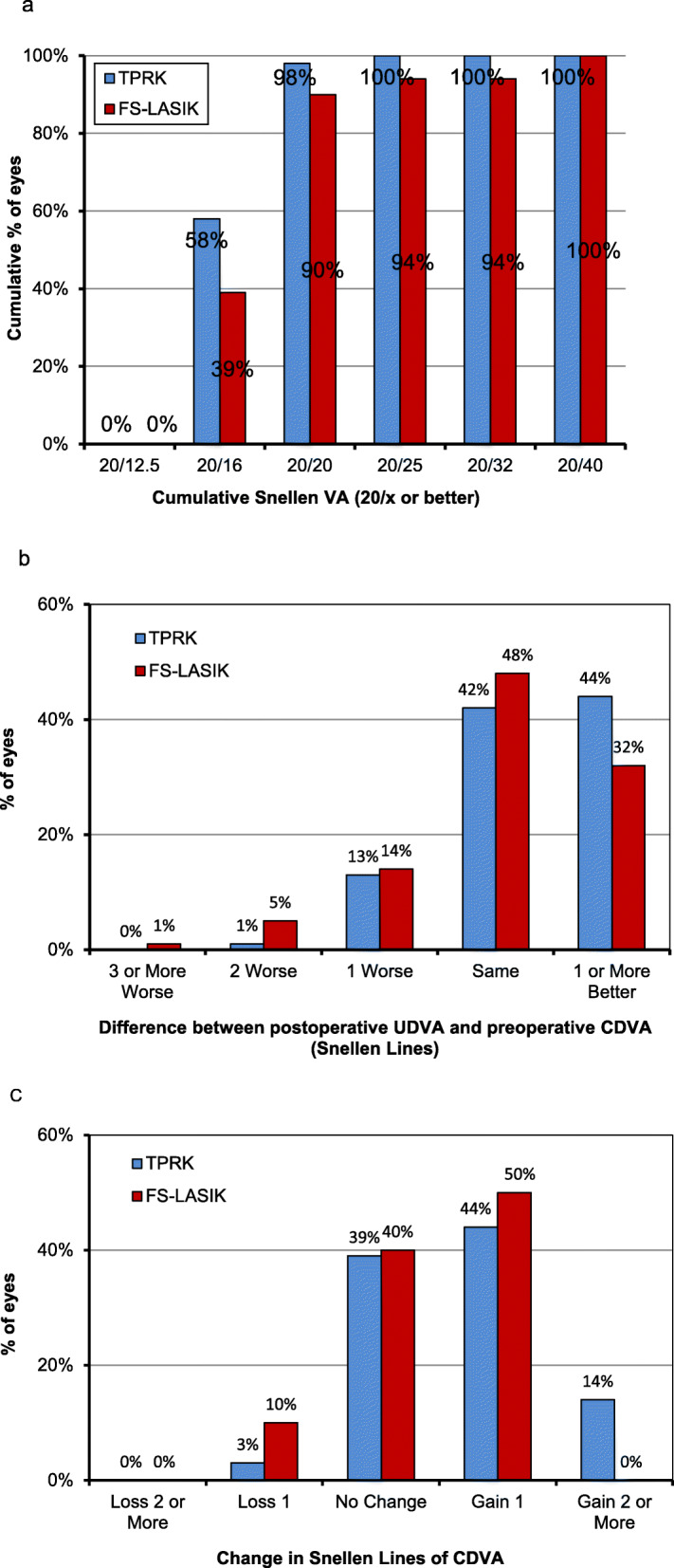
**a** Uncorrected distance visual acuity in TPRK and FS-LASIK group 12 months postoperatively. **b** Difference between uncorrected distance visual acuity postoperatively and corrected distance visual acuity preoperatively. **c** Change in corrected distance visual acuity between preoperation and 12-month postoperation

At postoperative 12 months, 98% of eyes in TPRK group and 90% of eyes in FS-LASIK group achieved 20/20 or better Snellen UDVA (*P* = 0.040, Fig. 1a). An UDVA of 20/32 or better was achieved 100% of eyes in the TPRK group and 94% in the FS-LASIK group (*P* = 0.025). At the last follow-up, the logMAR UDVA was − 0.04 ± 0.04 in the TPRK group and − 0.01 ± 0.08 in the FS-LASIK group (*P* = 0.039, Table 2). There were no significant differences for CDVA between the both groups (*P* = 0.621). For UDVA, 86% of eyes in TPRK group and 80% in FS-LASIK group remained no change or improved one or more lines (*P* = 0.314, Fig. 1b). For CDVA, 97% of eyes in TPRK group and 90% in FS-LASIK group remained no change or gained one or more lines (*P* = 0.096, Fig. 1c). No eye lost 2 lines or more in CDVA in either group. The calculated mean efficacy index (post UDVA/pre CDVA) in the two groups to be 1.06 and 1.01, and the safety index (post CDVA/pre CDVA) was 1.10 and 1.08, respectively, at postoperative 12 months.

**Table 2:** Postoperative visual acuity and refraction outcomes (mean ± SD)

Parameter	1 month	3 months	6 months	12 months	*P (time)*	*P (group)*
UDVA (log MAR)					0.87	0.039
TPRK	−0.03 ± 0.04	− 0.03 ± 0.04	− 0.05 ± 0.05	− 0.04 ± 0.04		
FS-LASIK	−0.03 ± 0.05	− 0.03 ± 0.05	− 0.02 ± 0.08	− 0.01 ± 0.08		
CDVA (log MAR)					0.075	0.621
TPRK	−0.03 ± 0.04	−0.03 ± 0.04	−0.06 ± 0.04	− 0.06 ± 0.05		
FS-LASIK	−0.04 ± 0.04	−0.04 ± 0.05	− 0.04 ± 0.04	−0.04 ± 0.05		
Sphere (D)					< 0.001	0.062
TPRK	0.31 ± 0.73	0.32 ± 0.43	0.16 ± 0.47	0.11 ± 0.39		
FS-LASIK	0.28 ± 0.50	0.19 ± 0.42	0.08 ± 0.54	−0.06 ± 0.47		
Cylinder (D)					0.511	0.511
TPRK	−0.40 ± 0.46	−0.33 ± 0.37	− 0.34 ± 0.25	−0.32 ± 0.20		
FS-LASIK	−0.39 ± 0.28	−0.35 ± 0.29	− 0.39 ± 0.29	−0.40 ± 0.34		
SER (D)					0.024	0.03
TPRK	0.11 ± 0.73	0.16 ± 0.46	−0.01 ± 0.48	− 0.05 ± 0.39		
FS-LASIK	0.08 ± 0.49	0.02 ± 0.41	−0.11 ± 0.54	−0.26 ± 0.47		

*UDVA* uncorrected distance visual acuity, *CDVA* corrected distance visual acuity, *SER* spherical equivalent refraction, *SD* standard deviation

### Refractive accuracy, predictability, stability

**Fig. 2:**
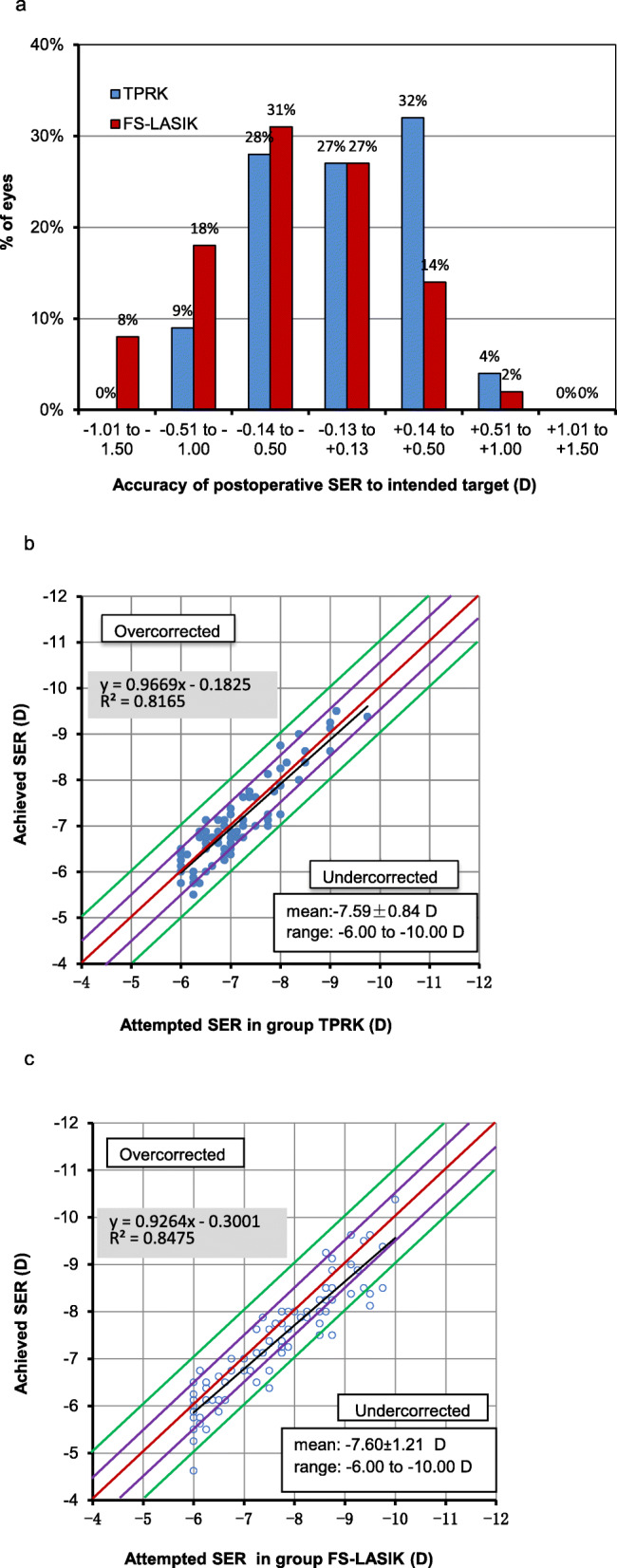
Percentages of eye of correction error in spherical equivalent refraction (SER) (postoperative SER subtracted intended target) 12 months after surgery (**a**). Relationship between attempted and achieved spherical equivalent refraction (SER) 12 months after surgery in group TPRK (**b**) and FS-LASIK (**c**)

**Fig. 3:**
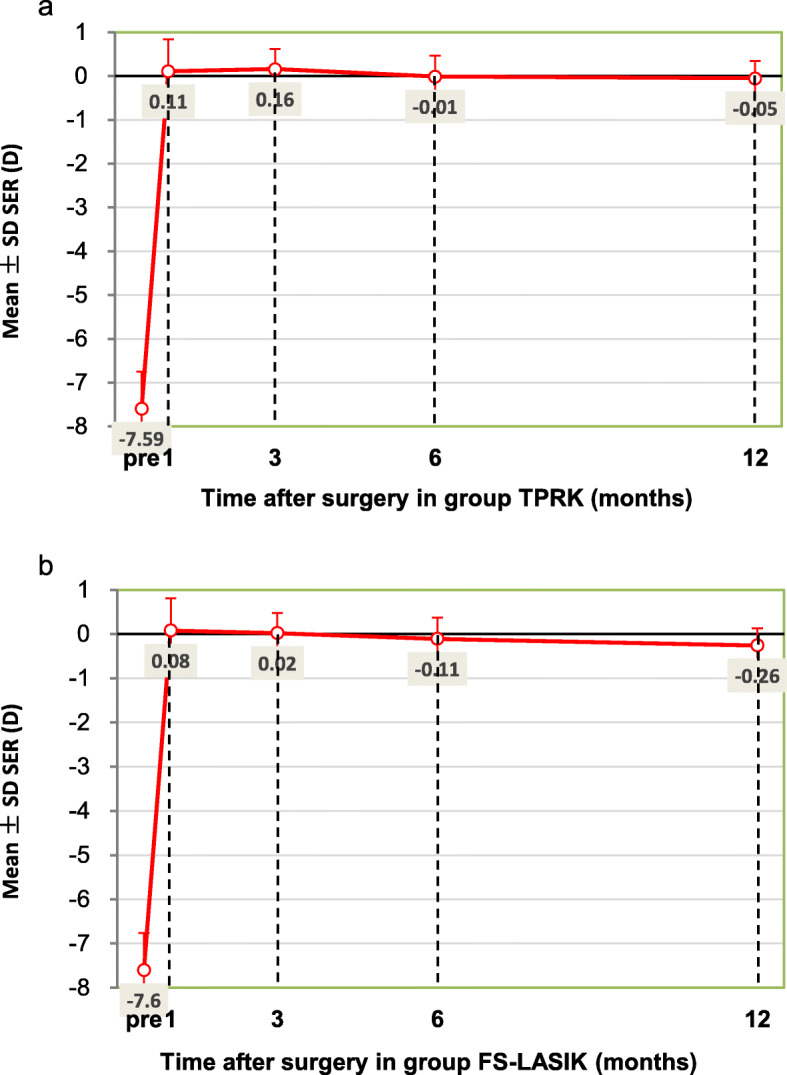
Spherical equivalent refraction (SER) stability during the 12-month follow-up in group TPRK (**a**) and FS-LASIK (**b**)

**Fig. 4:**
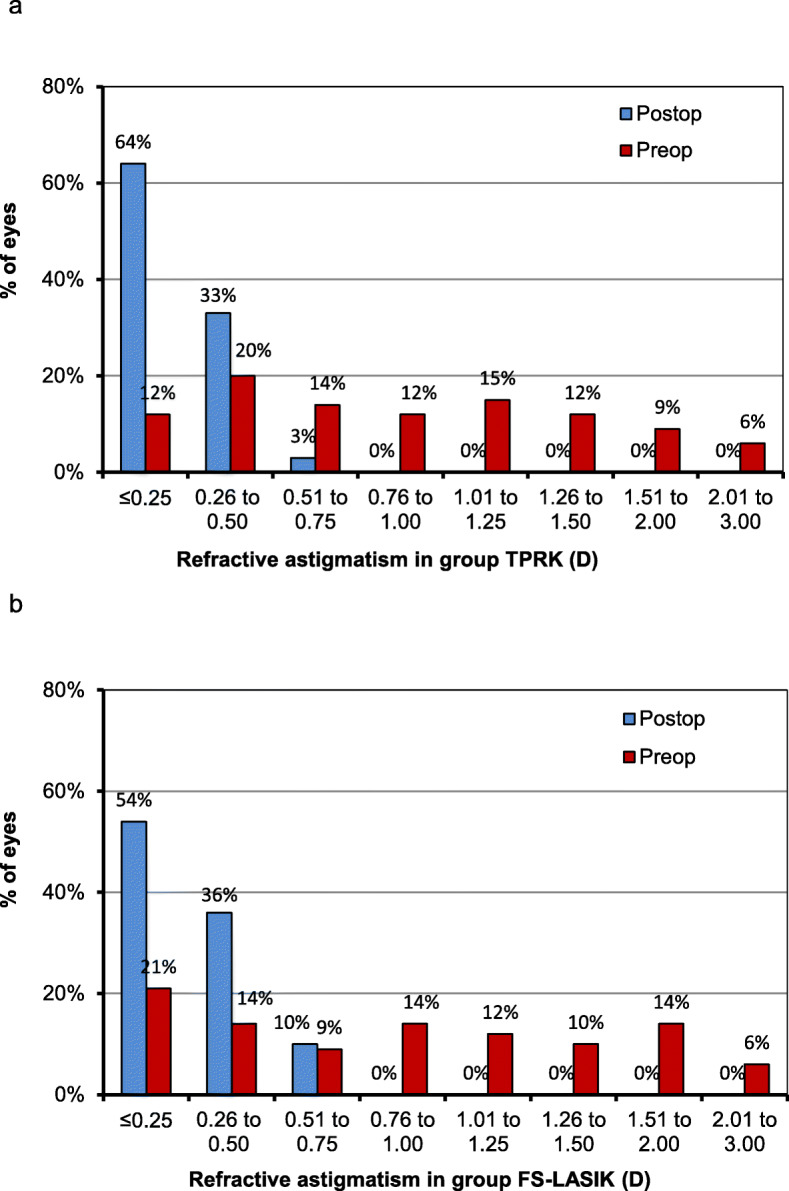
Changes of refractive astigmatism between preoperation and 12-month postoperation in group TPRK (**a**) and FS-LASIK (**b**)

At 12 months after surgery, mean spherical equivalent refraction was − 0.05 ± 0.39 D in the TPRK group and − 0.26 ± 0.47 D in the FS-LASIK group (*P* = 0.030, Table 2). 51% of eyes in the TPRK group and 45% in the FS-LASIK group achieved ±0.25 D target refraction (*P* = 0.473, Fig. 2a). There were significant differences for the target refraction of ±0.50 D, ±1.00 D between both groups (*P* = 0.019, *P* = 0.003, respectively). 87% of eyes in TPRK group and 73% in FS-LASIK group achieved ±0.50 D target refraction. The achieved refraction in all 85 eyes (100%) was ±1.00 D in the TPRK group and 74 eyes (92%) in the FS-LASIK group were ± 1.00 D. Linear regression analysis of achieved versus attempted SER for each group showed a coefficient (R^2^) of 0.8165 in the TPRK group (Fig. 2b), 0.8475 in the FS-LASIK group (Fig. 2c). A nearly linear relationship between achieved and attempted SER was shown in both groups. The refractive stability after surgery was shown in Fig. 3. Between 1-month and 12-month follow-up, number of eyes with more than 0.5 D change in SER were 10 (12%) and 14 (18%) in TPRK and FS-LASIK group, respectively. Refractive astigmatism in 97% of eyes for TPRK group and 90% of eyes for FS-LASIK group was within 0.50 D at postoperative 12 months (Fig. 4).

### High order aberration

The changes of corneal total HOA, horizontal and vertical coma, spherical aberration in both groups after surgery as compared to the preoperative data were shown in Tables 1 and 3. The root mean square (RMS) in total HOA, vertical coma and spherical aberration for the both groups increased after surgery. Only the horizontal coma remained stable from preoperation to postoperation. The RMS data of total HOA and vertical coma in the TPRK group was lower compared with the FS-LASIK group (*P* < 0.001, *P* < 0.001, respectively). There were no significant differences for the horizontal coma and spherical aberration between the both groups (*P* = 0.826, *P* = 0.102). At postoperative 12 months, RMS HOA were 1.05 ± 0.24 in the TPRK group and 1.29 ± 0.37 in the FS-LASIK group (*P* < 0.001), vertical coma were − 0.39 ± 0.39, − 0.65 ± 0.51 in both groups, respectively (*P* < 0.001).

**Table 3:** Postoperative high order aberration outcomes (mean ± SD)

Parameter	1 month	3 months	6 months	12 months	*P* (*time)*	*P (group)*
Corneal total HOA (μm)					0.09	< 0.001
TPRK	1.03 ± 0.23	1.01 ± 0.24	1.04 ± 0.23	1.05 ± 0.24		
FS-LASIK	1.29 ± 0.38	1.30 ± 0.43	1.30 ± 0.40	1.29 ± 0.37		
Spherical aberration (μm)					0.672	0.102
TPRK	0.68 ± 0.17	0.70 ± 0.18	0.71 ± 0.18	0.67 ± 0.17		
FS-LASIK	0.64 ± 0.22	0.65 ± 0.29	0.65 ± 0.21	0.63 ± 0.22		
Vertical coma (μm)					0.311	< 0.001
TPRK	−0.36 ± 0.38	−0.37 ± 0.37	−0.36 ± 0.39	−0.39 ± 0.39		
FS-LASIK	−0.65 ± 0.52	−0.65 ± 0.56	− 0.66 ± 0.53	−0.65 ± 0.51		
Horizontal coma (μm)					0.779	0.826
TPRK	−0.02 ± 0.36	−0.02 ± 0.36	− 0.02 ± 0.36	−0.03 ± 0.37		
FS-LASIK	−0.03 ± 0.55	−0.04 ± 0.57	− 0.04 ± 0.55	−0.03 ± 0.55		

*SD* standard deviation

## Discussion

Many studies had compared the efficacy and safety of LASIK or femtosecond laser-assisted LASIK with PRK or LASEK for correction of high myopia. Most showed that the visual outcomes of LASIK were better than PRK or LASEK. However, few studies have evaluated results of TPRK when treating high myopia, compared with LASIK or FS-LASIK. We compared clinical outcomes after surgery with TPRK and FS-LASIK for patients with high myopia. For preoperative variables in both groups, except for central corneal thickness (CCT), the other variables matched well. For only few patients with thinner CCT, we preferred to recommend patients to perform TPRK for the sake of biomechanical stability. So, the preoperative CCT in TPRK group was 7 μm less than that of FS-LASIK group (*P* = 0.050, close to statistical significance). It may be believed that difference of 7 μm in preoperative CCT had no significant effect on postoperatively clinical outcomes.

At postoperative 1 month, UDVA in TPRK group was equal to that in the FS-LASIK group. At final follow-up, UDVA was better in the TPRK group than the FS-LASIK group, whereas there was no difference in CDVA between groups. In our study, the efficacy and safety of both procedures was comparable, and no eyes showed 2 or more lines worse CDVA postoperatively, compared to preoperative CDVA. The postoperative UDVA and CDVA in both groups were acceptable, but when compared with other studies, percentage of UDVA achieving 20/40 or better was 95.4 to 100% in TPRK group [14–16] and the percentage was 88.2 to 99% in other FS-LASIK groups [17–20]. Comparisons of visual outcomes between TPRK and FS-LASIK group in our studies were similar to two others that described treatment for high myopia. Ghadhfan [16] found that in eyes with high myopia, transepithelial PRK provided better visual outcomes than LASIK, LASEK, or mechanical epithelial removal PRK. LASIK was associated with most major postoperative complications. Aslanides [15] found that TPRK for correction of high myopia demonstrated comparable refractive outcomes to LASIK and PRK, with relatively favorable visual acuity outcomes. Contrary to those studies and ours, Gershoni [20] reported that clinical outcomes of FS-LASIK were slightly better than those of TPRK. Another study compared the results of FS-LASIK and PRK for the correction of high myopia and found that FS-LASIK improved UDVA better than PRK [21].

Our efficacy and safety indices in both groups (all more than 1.00) were superior to those of previous studies that corrected myopia. Gershoni [20] reported that efficacy index values were 0.92 in their TPRK group and 0.95 in their FS-LASIK group. Corresponding safety index values were 0.95 and 0.97. Hashemi [22] found the efficacy indices of 0.99 and 0.93 in FS-LASIK and PRK group, respectively, and safety indices of 1.01 and 1.01, respectively. The difference in outcomes may be due to the smart pulse technology used by Amaris 750S excimer laser, which improved residual bed smoothness and reduced irregularity. Vinciguerra [23] found that excimer laser coupled with smart pulse technology led to improvement of 6-month uncorrected visual acuity, compared with use of conventional techniques. The smart pulse technology software features a particular characterization of the ablative spot geometry. This avoids the thermal load and ablation effect of pulse energy not effectively applied in the ablation process [24].

For surface ablation, the use of mitomycin could surely reduce the possibility of haze formation, especially when correcting high myopia. However, in our study we did not use mitomycin in TPRK group. There was no haze above 0.5 level in the TPRK group at the last follow-up. We assumed that it was due to improved surface smoothness and less ineffective energy invested in the ablation process induced by the smart pulse technology. After laser ablation, using cold balanced salt solution is also beneficial, because it could lessen the thermal effect of laser ablation, which in turn decreases formation of corneal haze. Furthermore, after surgery all patients were treated with 0.1% fluoromethane drops for 4 months and strictly followed up every month. If necessary, short-term 1% prednisone acetate drops were used to inhibit haze formation. All patients were also required to wear sunglasses to prevent ultraviolet rays which could increase the incidence of haze.

At postoperative 12 months, we found refractive predictability to be higher in the TPRK group than the FS-LASIK group. Postoperative refraction was never more than ±1.00 D in the TPRK group. Six eyes were outside ±1.00 D in the FS-LASIK group, where UDVA was decreased. The percentage of eyes within ±0.50D was higher than that in Gershoni’s study [20], but lower than that in Aslanides’ study [15]. Refractive stability was also better in the TPRK group. During postoperative follow-up, the spherical equivalent refraction changed from + 0.11 D to − 0.05 D in the TPRK group and + 0.08 D to − 0.26 D in the FS-LASIK group. At 12 months, refractive status was more minus in the FS-LASIK group than the TPRK group, with some undercorrection observed in the FS-LASIK group. In Luger’s study [25], small overcorrection was also observed in the eyes that had TPRK, and some undercorrection was observed in the eyes that had FS-LASIK. Better UDVA in the TPRK group might have occurred because of slight overcorrection. Unlike spherical refraction, changes in refractive astigmatism were similar in both groups. The large reduction in astigmatism at postoperative 1 month was similar in both groups and remained unchanged until postoperative 12 months. TPRK and FS-LASIK were both efficient and relatively safe procedures for the correction of astigmatism.

In this study, the corneal RMS HOA increased after surgery in both groups, the amount of change was larger in the FS-LASIK group at 12 months postoperative. Another difference was vertical coma values, which were higher in the FS-LASIK group, compared with the TPRK group. Changes of spherical aberration and horizontal coma were similar in both groups. There was a corneal flap created in the FS-LASIK group, but there was no incision in the TPRK group, so corneal integrity was maintained. The flap made with the hinge at the superior location in this study may explain why the vertical coma and corneal HOA were higher in the FS-LASIK group. Similar findings [22, 26–28] reported that horizontal coma was induced when the flap was made on the nasal side and vertical coma was induced with the flap at the superior location.

The main limitations of our study were a relatively short-term follow-up of 12 months and some omission of visual quality data (such as contrast sensitivity). A longer follow-up period is necessary for comprehensive evaluation of visual acuity. Although we found that HOA changed, especially vertical coma and spherical aberration, it remained unclear how HOA increase influenced quality of vision. HOA was related to perception of shadows, halos and night vision glare, and a reduction in contrast sensitivity. Visual quality might better reflect the clinical outcomes compared with visual acuity, but a reliable metric for quality must be established. We believed that due to no haze formation and smaller coma, the contrast sensitivity of TPRK group may be better than that of FS-LASIK group. This needs further research to confirm.

## Conclusions

The current study found that TPRK and FS-LASIK is safe, efficacious, and predictable for correction of high myopia. TPRK are slightly better than FS-LASIK.

## Data Availability

The datasets used and/or analysed during the current study are available from the corresponding author on reasonable request.

## Abbreviations

TPRK: Transepithelial photorefractive keratectomy
FS-LASIK: Femtosecond laser-assisted in situ keratomileusis
UDVA: Uncorrected distance visual acuity
CDVA: Corrected distance visual acuity
SER: Spherical equivalent refraction
HOAs: Higher order aberrations
